# Benefits observed in an inpatient MDT programme for FND are not associated with medication use or previous therapies

**DOI:** 10.1192/bjo.2021.112

**Published:** 2021-06-18

**Authors:** Thomas Elliott, Michael Elmalem

**Affiliations:** 1 Camden and Islington NHS Foundation Trust; 2 National Hospital for Neurology and Neurosurgery

## Abstract

**Aims:**

The National Hospital for Neurology and Neurosurgery provides various services for patients with Functional Neurological Disorder (FND), including a four-week inpatient rehabilitation programme run by an integrated Multi-Disciplinary Team (MDT) of Occupational Therapists (OT), Physiotherapists (PT), Psychologists and Psychiatrists.

We had observed that patients with FND often have medical and psychiatric comorbidities including affective, dissociative, somatic symptom and pain disorders; pharmacological treatments are commonly used. We hypothesised that a high burden of medication, particularly of those which produce dependence, might limit one's ability to entrain therapeutic strategies and therefore benefit from treatment. We additionally hypothesised that patients who had previously tried individual physical or psychological therapies might gain less than those who were treatment-naïve.

**Method:**

In this service evaluation project, we reviewed records from 97 consecutive elective inpatient admissions, comprising the entire intake for 2019 and 2020. Data were extracted from the inpatient discharge summary and therapies discharge report of each patient. We recorded which therapies for FND patients had previously tried (OT; PT; Speech and Language Therapy; Psychology; Pain Service) and the classes of medications they were taking on admission (opiates; benzodiazepines; antidepressants; mood stabilisers; antipsychotics; gabapentinoids). We compared the differentials in outcome measures recorded on the first and last day, including the Canadian Occupational Performance Measure (n = 79) and EQ-5D-5L (n = 79). Statistical tests of effect size and significance were done using SPSS-25. Group comparisons of EQ-5D-5L were made with Paired t-tests; all other comparisons were done with Wilcoxon signed-rank tests due to non-normal data.

**Result:**

The most common medications used were antidepressants (72%), gabapentinoids (39%), opiates (36%) and benzodiazepines (25%). 69% of patients had tried PT, 57% psychology and 52% OT, while only 13% were treatment naïve. Whole-cohort analysis revealed highly significant improvements (p < 0.001) in occupational performance, satisfaction, ratings of general health, subjective difficulty in performing tasks and in pain and fatigue levels. We found no significant differences in outcome measures that correlated with past therapies or medication use.

**Conclusion:**

Our analysis shows that the great majority of our patients gained meaningful benefits from their admission, both on clinician-rated metrics of occupational performance and patient-rated measures of subjective improvement. That there was no significant relationship with therapies or medications suggests, promisingly, that patients taking various medications and with suboptimal responses to previous therapy can still benefit from our MDT programme. Limitations include correlational design, limited generalisability to the general population, missing data for certain outcome measures and the absence of follow-up data.

